# Association between lactate/albumin ratio and all-cause mortality in critical patients with acute myocardial infarction

**DOI:** 10.1038/s41598-023-42330-8

**Published:** 2023-09-20

**Authors:** Danni Wang, Chaodi Luo, Qian Li, Tingting Zheng, Pengjie Gao, Boxiang Wang, Zhenzhen Duan

**Affiliations:** 1 Department of Cardiology, First Affiliated Hospital of Xi’an Jiaotong University, Xi’an, People’s Republic of China; 2 Department of Peripheral Vascular Diseases, Honghui Hospital of Xi’an Jiaotong University, 555 Youyi East Road, Xi’an, People’s Republic of China

**Keywords:** Biomarkers, Cardiology, Diseases, Health care, Medical research, Risk factors

## Abstract

It has been demonstrated that lactate/albumin (L/A) ratio is substantially relevant to the prognosis of sepsis, septic shock, and heart failure. However, there is still debate regarding the connection between the L/A ratio and severe acute myocardial infarction (AMI). The aim of this study is to determine the prognostic role of L/A ratio in patients with severe AMI. Our retrospective study extracted data from the Medical Information Mart for Intensive Care III (MIMIC-III) database, included 1,134 patients diagnosed with AMI. Based on the tertiles of L/A ratio, the patients were divided into three groups: Tertile1 (T1) group (L/A ratio<0.4063, *n*=379), Tertile2 (T2) group (0.4063≤L/A ratio≤0.6667, *n* =379), and Tertile3 (T3) group (L/A ratio>0.6667, *n* =376). Uni- and multivariate COX regression model were used to analyze the relationship between L/A ratio and 14-day, 28-day and 90-day all-cause mortality. Meanwhile, the restricted cubic spline (RCS) model was used to evaluate the effect of L/A ratio as a continuous variable. Higher mortality was observed in AMI patients with higher L/A ratio. Multivariate Cox proportional risk model validated the independent association of L/A ratio with 14-day all-cause mortality [hazard ratio (HR) 1.813, 95% confidence interval (CI) 1.041-3.156 (T3 vs T1 group)], 28-day all-cause mortality [HR 1.725, 95% CI 1.035-2.874 (T2 vs T1 group), HR 1.991, 95% CI 1.214-3.266 (T3 vs T1 group)], as well as 90-day all-cause mortality [HR 1.934, 95% CI 1.176-3.183 (T2 vs T1 group), HR 2.307, 95% CI 1.426-3.733 (T3 vs T1 group)]. There was a consistent trend in subgroup analysis. The Kaplan-Meier (K-M) survival curves indicated that patients with L/A ratio>0.6667 had the highest mortality. Even after adjusting the confounding factors, RCS curves revealed a nearly linearity between L/A ratio and 14-day, 28-day and 90-day all-cause mortality. Meanwhile, the areas under the receiver operating characteristic (ROC) curve (AUC) of 14-day, 28-day and 90-day all-cause mortality were 0.730, 0.725 and 0.730, respectively. L/A ratio was significantly associated with 14-day, 28-day and 90-day all-cause mortality in critical patients with AMI. Higher L/A ratio will be considered an independent risk factor for higher mortality in AMI patients.

## Introduction

Cardiovascular disease remains the leading cause of death worldwide^1^. Acute myocardial infarction (AMI), as a common serious coronary event may develop complications, including heart failure, arrhythmia, cardiogenic shock, cardiac arrest and other serious adverse outcomes^2, 3,4^. Despite the advanced treatment strategies, such as percutaneous coronary intervention (PCI) and coronary artery bypass grafting (CABG), the mortality rate of AMI varies globally, resulting in a great health burden^5, 6,7^. To provide more clinical information, a growing number of indicators are explored to forecast the prognosis of AMI patients.

Lactate, a familiar indicator often used to predict the prognosis of disease and the severity of shock^8^, has been shown to be associated with mortality from a variety of diseases^9,10, 11^. A previous study involving 1,176 patients with ST-segment elevation myocardial infarction found that elevated lactate levels were associated with increased 1-day acute mortality and increased 30-day mortality^12^. Albumin is an available and inexpensive clinical indicator. In addition to judging liver function and nutritional status, it has been demonstrated that cardiovascular disease incidence and hypoalbuminemia are closely connected. Hypoalbuminemia is an independent prognostic factor and modifiable risk factor that deserves attention for many cardiovascular diseases^13^. A retrospective study indicated that patients with low serum albumin on admission had a poor prognosis^14^. Therefore, both of the two indicators have important potential roles in a variety of diseases, especially for cardiovascular diseases. Recently, as a straightforward and simple-to-obtain composite indicator, the lactate/albumin (L/A) ratio has been proposed. Currently, most studies focus on its prognostic effect on sepsis patients^15,16–18^. Some studies have shown that L/A ratio has higher predictive value than lactate or albumin in mortality of sepsis patients^15, 16^. In addition to sepsis, studies also have found that the L/A ratio is related to both short- and long-term mortality in individuals who suffered from heart failure driven on by AMI^19, 20^.

However, it is still unclear whether lactate/albumin ratio and all-cause mortality in critical patients with AMI are relevant. Therefore, the aim of our study is to evaluate the predictive value of L/A ratio for mortality after AMI.

## Results

### Baseline characteristics of the study population

A total of 1,134 patients were included in our analysis. The average age of which was 68.87 and the percentage of men was 67.9%. Among all individuals, there were 145(12.8%) all-cause death within 14 days, 174 (15.3%) all-cause death within 28 days and 186 (16.4%) all-cause death within 90 days after intensive care unit (ICU) admission. A group analysis based on the tertiles of lactate/albumin ratio were shown in Table 1. The patients in Tertile3 (T3) group were older and more female, higher proportion of patients suffering from atrial fibrillation (AF), acute kidney injury (AKI), acute respiratory distress syndrome (ARDS) and sepsis, lower proportion of patients with hypertension and hyperlipidemia, and fewer patients using aspirin, beta blockers, diuretics, statin, insulin, and oral hypoglycemic agents. With increasing L/A ratio, heart rate, respiratory rate, base excess (BE), anion gap (AG), lactate, white blood cell (WBC), blood urea nitrogen (BUN), prothrombin time (PT) increased, systolic blood pressure (SBP) and albumin decreased. As L/A ratio increased, scores from the Simplified Acute Physiology Score II (SAPS II) and sequential organ failure assessment (SOFA) became greater. Higher L/A ratio were substantially correlated with higher 14-day, 28-day and 90-day all-cause mortality, cardiac arrest and cardiac shock and less invasive ventilation. However, there was no significant difference among the three groups regarding the type of coronary artery revascularization after admission.

**Table 1:** Baseline characteristics across L/A ratio.

Variable	Overall N=1134	L/A ratio < 0.4063 *n*=379	0.4063≤L/A ratio≤ 0.6667 *n*=379	L/A ratio > 0.6667 *n*=376	p value
Age, years	68.87 (60.74–77.22)	67.16 (58.90–75.45)	68.63 (60.73–77.68)	70.89 (62.13–77.66)	0.004
Male, n (%)	770 (67.9)	274 (72.3)	260 (68.6)	236 (62.8)	0.018
Ethnicity, n (%)					0.678
Asian	27 (2.4)	8 (2.1)	11 (2.9)	8 (2.1)	
Black	50 (4.4)	13 (3.4)	16 (4.2)	21 (5.6)	
White	802 (70.7)	274 (72.3)	273 (72.0)	255 (67.8)	
Hispanic or Latino	29 (2.6)	12 (3.2)	8 (2.1)	9 (2.4)	
other	31 (2.7)	13 (3.4)	7 (1.8)	11 (2.9)	
unknown	195 (17.2)	59 (15.6)	64 (16.9)	72 (19.1)	
Types of hospital admission, n (%)					0.137
Elective	235 (20.7)	89 (23.50)	76 (20.10)	70 (18.6)	
Emergency	855 (75.4)	280 (73.9)	290 (76.5)	285 (75.8)	
Urgent	44 (3.9)	10 (2.60)	13 (3.40)	21 (5.6)	
Past medical history, n (%)					
AF	425 (37.5)	125 (33.0)	143 (37.7)	157 (41.8)	0.045
Hypertension	572 (50.4)	201 (53.0)	201 (53.0)	170 (45.2)	0.046
Diabetes	403 (35.5)	135 (35.6)	140 (36.9)	128 (34.0)	0.707
Hyperlipidemia	190 (16.8)	83 (21.9)	58 (15.3)	49 (13.0)	0.003
COPD	18 (1.6)	7 (1.8)	5 (1.3)	6 (1.6)	0.844
AKI	273 (24.1)	70 (18.5)	91 (24.0)	112 (29.8)	0.001
ARDS	100 (8.8)	18 (4.7)	34 (9.0)	48 (12.8)	0.001
SEPSIS	73 (6.4)	12 (3.2)	27 (7.1)	34 (9.0)	0.004
CHF	418 (36.9)	134 (35.4)	136 (35.9)	148 (39.4)	0.464
Vital signs					
SBP, mmHg	110.35 (102.43–119.08)	111.81 (103.87–121.87)	111.45 (103.75–120.27)	106.79 (99.38–115.92)	<0.001
DBP, mmHg	57.45 (52.55–63.32)	57.92 (53.17–64.09)	58.30 (52.70–63.24)	56.22 (51.41–62.09)	<0.001
MBP, mmHg	74.70 (70.07–80.43)	74.77 (70.39–81.09)	75.79 (71.22–80.86)	74.07 (68.99–79.40)	0.002
HR, beats/min	84.83 (77.18–93.23)	83.16 (76.32–90.07)	85.37 (77.88–93.09)	86.62 (77.51–96.81)	0.004
RR, times/min	17.82 (15.83–20.48)	17.67 (15.75–19.95)	17.64 (15.79–20.22)	18.15 (15.95–21.52)	0.035
T, ℃	36.84 (36.46–37.28)	36.82 (36.50–37.28)	36.90 (36.52–37.31)	36.83 (36.37–37.25)	0.178
Laboratory data					
pH	7.39 (7.34–7.43)	7.39 (7.34–7.43)	7.39 (7.35–7.43)	7.39 (7.33–7.44)	0.879
SpO2, %	97.93 (96.61–98.97)	97.71 (96.57–98.87)	98.15 (96.72–99.04)	97.83 (96.50–98.87)	0.139
BE, mmol/L	0 (–3–1)	0 (–2–2)	0 (–3–1)	−1 (−5−1)	<0.001
AG, mmol/L	13 (12−16)	13 (11−15)	13 (11−15)	14 (12−17)	<0.001
Lactate, mmol/L	1.70 (1.20−2.60)	1.10 (0.90−1.30)	1.80 (1.50−2.00)	3.10 (2.50−4.40)	<0.001
Hemoglobin, g/dL	10.60 (9.40−11.90)	10.60 (9.40−12.10)	10.70 (9.40−11.90)	10.50 (9.30−11.90)	0.907
PLT, K/uL	204.50 (149−285)	208 (153−282)	199 (156−282)	203.50 (133−294.25)	0.409
WBC, K/uL	10.40 (7.80−13.70)	9.70 (7.30−12.60)	10.50 (7.90−13.70)	11.20 (8.13−14.98)	<0.001
ALB, g/dL	3.40 (2.80−3.90)	3.80 (3.30−4.10)	3.40 (2.80−3.90)	3.00 (2.43−3.60)	<0.001
BUN, mg/dL	21 (15−34)	19 (14−31)	20 (15−32)	24 (17.25−41)	<0.001
Scr, mg/dL	1.10 (0.80−1.53)	1 (0.80−1.50)	1 (0.8−1.5)	1.1 (0.8−1.7)	0.052
Glucose, g/dL	126 (104−161)	126 (103−151)	124.50 (104−164.25)	129 (104.25−171.75)	0.115
Sodium, mEq/L	138.14 (136.25–139.82)	138.14 (136.38–139.60)	138.06 (136.21–139.92)	138.20 (136.12–140.25)	0.791
Potassium, mEq/L	4.15 (3.80–4.50)	4.20 (3.8–4.5)	4.10 (3.70–4.50)	4.20 (3.80–4.60)	0.268
PT, s	13.90 (13.00–15.63)	13.70 (12.80–15.30)	14 (13–15.5)	14.10 (13.13–16.28)	0.009
PTA, %	33.7 (27.93–47.93)	32.80 (27.20–47.20)	34.00 (28.25–44.65)	35.20 (28.50–50.18)	0.055
Oral medication					
Aspirin, n (%)	979 (86.3)	352 (92.9)	322 (85.0)	305 (81.1)	<0.001
Clopidogrel, n (%)	450 (39.7)	150 (39.6)	139 (36.7)	161 (42.8)	0.225
Beta blockers, n (%)	901 (79.5)	334 (88.1)	314 (82.8)	253 (67.3)	<0.001
Diuretics, n (%)	853 (75.2)	306 (80.7)	292 (77.0)	255 (67.8)	<0.001
Digitalis, n (%)	73 (6.4)	18 (4.7)	32 (8.4)	23 (6.1)	0.111
Statin, n (%)	866 (76.4)	326 (86.0)	297 (78.4)	243 (64.6)	<0.001
Anti-diabetic therapy					
Insulin, n(%)	904 (79.7%)	311 (82.1)	309 (81.5)	284 (75.5)	0.047
Oral hypoglycemic agents, n(%)	112 (9.9)	43 (11.4)	48 (12.7)	21 (5.6)	0.002
Coronary artery revascularization					0.346
PCI, n(%)	115 (10.10)	27 (7.1)	42 (11.1)	46 (12.2)	
PTCA, n(%)	31 (2.7)	10 (2.6)	12 (3.2)	9 (2.4)	
CABG, n(%)	462 (40.7)	166 (43.8)	164 (43.3)	132 (35.1)	
PCI+PTCA, n(%)	118 (10.4)	45 (11.9)	30 (7.9)	43 (11.4)	
PCI+CABG, n(%)	6 (0.5)	2 (0.5)	2 (0.5)	2 (0.5)	
PTCA+CABG, n(%)	14 (1.2)	5 (1.3)	4 (1.1)	5 (1.3)	
PCI+PTCA+CABG, n(%)	9 (0.8)	2 (0.5)	3 (0.8)	4 (1.1)	
Score data					
SOFA	5 (3–7)	4 (2–7)	4 (3–7)	6 (4–9)	<0.001
SAPSII	37 (30–48)	35 (28–43)	36 (30–45)	44 (33–54)	<0.001
Clinical outcome, n (%)					
14 days all-cause mortality	145 (12.8)	19 (5.0)	40 (10.6)	86 (22.9)	<0.001
28 days all-cause mortality	174 (15.3)	23 (6.1)	51 (13.5)	100 (26.6)	<0.001
90 days all-cause mortality	186 (16.4)	24 (6.3)	55 (14.5)	107 (28.5)	<0.001
cardiac arrest	98 (8.6)	19 (5.0)	25 (6.6)	54 (14.4)	<0.001
Cardiac shock	197 (17.4)	44 (11.6)	55 (14.5)	98 (26.1)	<0.001
Noninvasive ventilation	43 (3.8)	21 (5.5)	12 (3.2)	10 (2.7)	0.086
Invasive ventilation	367 (32.4)	145 (38.3)	132 (34.8)	90 (23.9)	<0.001

Continuous variables that conform to the normal distribution are expressed as mean ± standard deviation. Continuous variables that are not normally distributed are expressed as median (interquartile range). Categorical variables are expressed as numbers (percentages). AF, atrial fibrillation; COPD, chronic obstructive pulmonary disease; AKI, acute kidney injury; ARDS, acute respiratory distress syndrome; CHF, congestive heart failure; SBP, systolic blood pressure; DBP, diastolic blood pressure; MBP, mean blood pressure; HR, heart rate; RR, respiratory rate;T, temperature; pH, potential of hydrogen; SpO2, saturation of hemoglobin with oxygen; BE, base excess; AG, anion gap; PLT, platelet; WBC, white blood cell; ALB, albumin; BUN, blood urea nitrogen; SCr, serum creatinine; PT, prothrombin time; PTA, prothrombin time activity; PCI, percutaneous coronary intervention; PTCA, percutaneous transluminal coronary angioplasty; CABG, coronary artery bypass graft; SOFA, sequential organ failure assessment; SAPS II, the Simplified Acute Physiology Score II.

### The association between the L/A ratio and AMI

The outcomes of the Cox proportional-risk model for 28-day all-cause mortality were displayed in Table 2. In the unadjusted Cox model, Tertile2 (T2) and T3 groups had greater 28-day all-cause mortality compared to Tertile1 (T1) group [hazard ratio (HR) 2.266, 95% confidence interval (CI) 1.385-3.708 for T2; HR 4.970, 95% CI 3.158-7.821 for T3]. After adjusting for confounding factors, 28-day risk of death was 1.725-fold (HR 1.725, 95% CI 1.035-2.874) higher in T2 and 1.991-fold (HR 1.991, 95% CI 1.214-3.266) higher in T3 than T1. More results were observed for 14-day all-cause mortality (Supplementary Table 1). After adjusting for confounding factors, 14-day mortality was still statistically higher in T3 than in T1(HR 1.813, 95% CI 1.041-3.156). It is concluded that higher L/A ratio (>0.6667) is an independent risk factor for short-term 14-day and 28-day all-cause mortality to AMI patients. At the same time, we further studied the relationship between L/A ratio and 90-day all-cause mortality (Supplementary Table 2), and the similar results have been achieved.

**Table 2:** Cox proportional hazard models for 28-day all-cause death.

Variables	L/A ratio < 0.4063	0.4063≤L/A ratio≤ 0.6667	L/A ratio > 0.6667
Model 1^a^	1.000 (Ref.)	2.266 (1.385–3.708)	4.970 (3.158–7.821)
P value	–	0.001	<0.001
Model 2^b^	1.000 (Ref.)	2.233 (1.351–3.691)	4.259 (2.668–6.796)
P value	–	0.002	<0.001
Model 3^c^	1.000 (Ref.)	2.198 (1.329–3.635)	4.096 (2.564–6.544)
P value	–	0.012	<0.001
Model 4^d^	1.000 (Ref.)	1.794 (1.082–2.974)	2.720 (1.688–4.385)
P value	–	0.024	<0.001
Model 5^e^	1.000 (Ref.)	1.725 (1.035–2.874)	1.991 (1.214–3.266)
P value	–	0.036	0.006

^a^ Model 1 Univariate model.

^b^Model 2 adjusted for age, gender, SBP, DBP.

^c^Model 3 adjusted for model 2 plus hypertension, diabetes, hyperlipemia, AF, COPD, CHF.

^d^Model 4 adjusted for model 3 plus aspirin, clopidogrel, beta blockers, diuretics, digitalis, statin, insulin, oral hypoglycemic agents.

^e^Model 5 adjusted for model 4 plus BUN, Scr, glucose, WBC, Hb, BE, SpO_2_.

**Figure 1:**
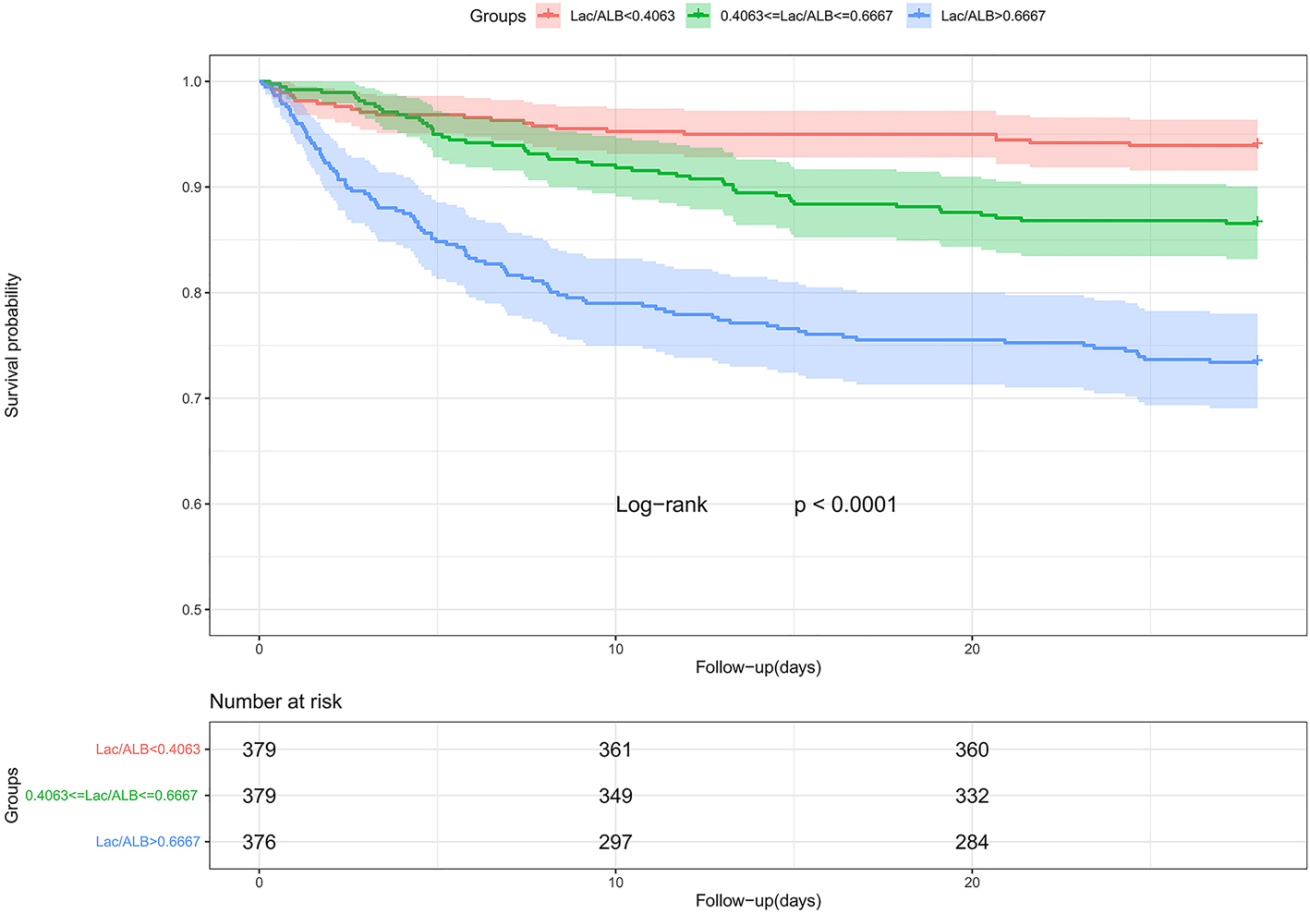
Kaplan-Meier survival curve of 28-day all-cause mortality stratified by L/A ratio.

**Figure 2:**
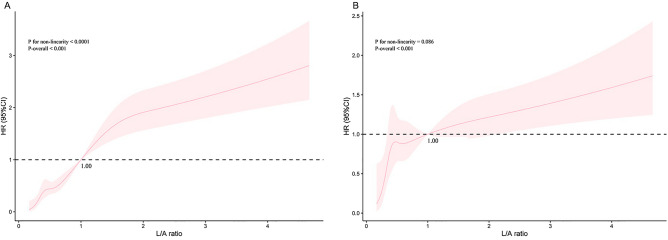
The adjusted cubic spline model on the association between L/A ratio on a continuous scale and adjusted risk of 28-day all-cause mortality in patients with AMI. Crude hazard ratio (HR) and 95% CI for L/A ratio in 28-day mortality(A). Adjusted HR and 95% CI for L/A ratio in 28-day mortality (B). Adjusted variables included age, gender, SBP, DBP, hypertension, diabetes, hyperlipemia, AF, COPD, CHF, aspirin, clopidogrel, beta blockers, diuretics, digitalis, statin, insulin, oral hypoglycemic agents, BUN, Scr, glucose, WBC, Hb, BE, SpO_2_.

The Kaplan-Meier (K-M) survival curve analysis revealed statistical differences in 28-day mortality among the three groups by L/A ratio tertiles. Compared to the T2 and T1 groups (Fig. 1), the T3 group had substantially greater 28-day all-cause mortality (*p*<0.001). Similar results were found in K-M analysis of 14-day and 90-day all-cause mortality (Supplementary Figure 1). According to restricted cubic spline (RCS) analysis, 28-day all-cause mortality risk rose non-linearly of increasing L/A ratio in unadjusted models (P for non-linear <0.001), whereas a nearly linear association existed between L/A ratio and the risk of 28-day mortality in models adjusted for age, gender, SBP, diastolic blood pressure (DBP), hypertension, diabetes, hyperlipemia, AF, chronic obstructive pulmonary disease (COPD), congestive heart failure (CHF), aspirin, clopidogrel, beta blockers, diuretics, digitalis, statin, insulin, oral hypoglycemic agents, BUN, serum creatinine (Scr), glucose, WBC, hemoglobin (Hb), BE, saturation of hemoglobin with oxygen (SpO_2_) (P for non-linearity =0.086) (Fig. 2). In addition, we found that the L/A ratio also had a nearly linear relationship with all-cause mortality at 14 and 90 days (Supplementary Figure 2 and Supplementary Figure 3).

**Figure 3:**
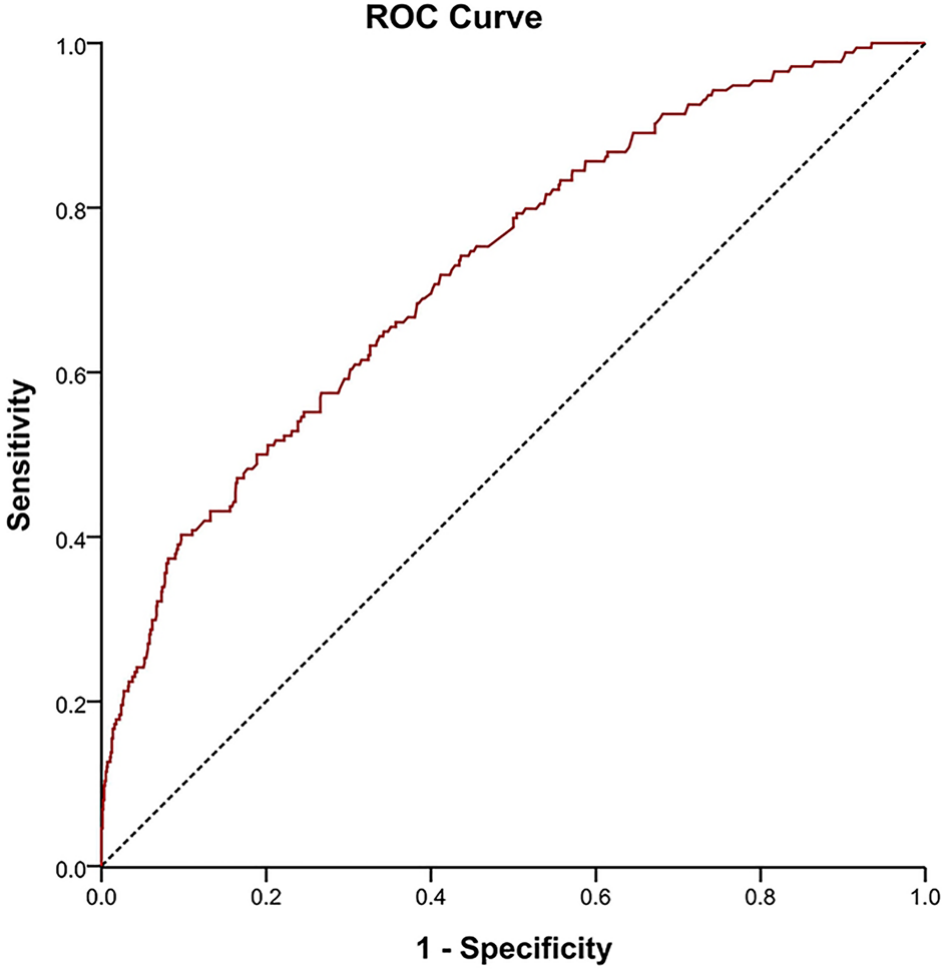
Receiver operating characteristic (ROC) curve for 28-day all-cause mortality.

The area under the receiver operating characteristic (ROC) curve of the L/A ratio for 28-day all-cause mortality was shown in Fig. 3. Meanwhile, Supplementary Figure 4 and 5also showed similar ROC curves for 14-day and 90-day all-cause mortality. The area under the ROC curve (AUC) manifested that the L/A ratio was able to predict mortality in AMI patients (The AUCs for 14-day, 28-day and 90-day all-cause mortality were 0.730, 0.725, and 0.730, respectively).

### Subgroup analysis

**Figure 4:**
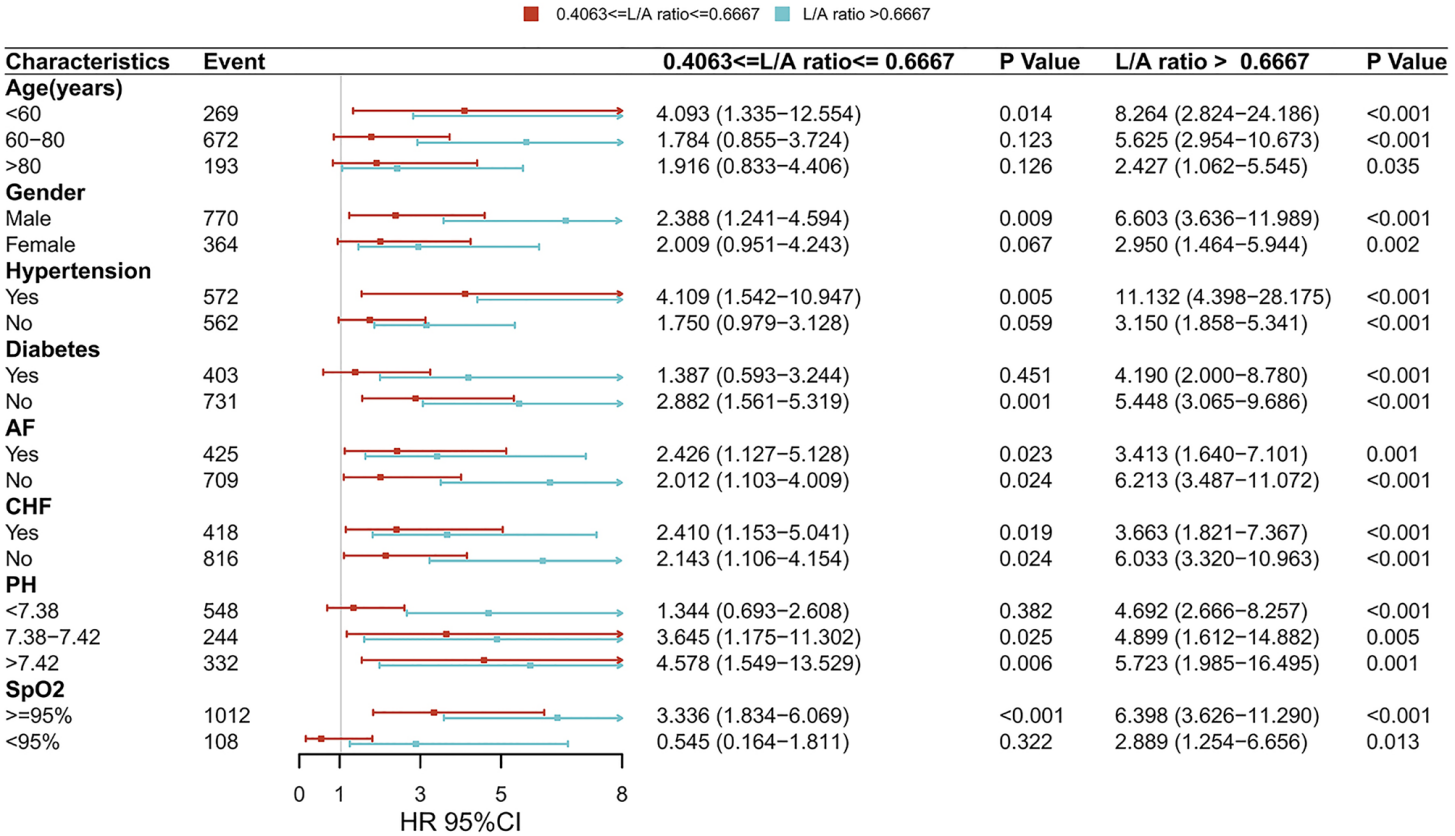
Association between L/A ratio and risk of all-cause mortality in subgroups. Forest plot and adjusted HR with 95% CI for 28-day all-cause mortality.

To further explore whether the association might vary under multiple conditions, we performed a subgroup analysis of age, gender, hypertension, diabetes, AF, CHF, potential of hydrogen (pH), and SpO_2_ (Fig. 4). Interestingly, within all subgroups, high level of L/A ratio had higher 28-day all-cause mortality. These results suggested that the L/A ratio has good predictive value for AMI patients.

## Discussion

In our study, we discovered that in critical AMI patients, L/A ratio was substantially relevant to 14-day, 28-day and 90-day all-cause mortality. The correlation of L/A ratio with all-cause mortality remained stable after adjusting for confounders and also significant in subgroup analysis. Moreover, RCS analysis indicated that L/A ratio and all-cause mortality were found to be nearly linearly correlated and the cut-off value is 1.00. In addition, the AUCs of the L/A ratio for predicting 14-day, 28-day and 90-day all-cause mortality in AMI patients were 0.730, 0.725 and 0.730. These results indicated that L/A ratio was capable of serving as a better predictive marker of prognosis in AMI clinical practice.

The clinical significance of lactate has been raised since its discovery in 1780 and its introduction into medicine in 1843^22, 23^. Studies have shown that elevated serum lactate levels can reflect reduced systemic blood flow, tissue hypoxia, and hypoperfusion^24, 25^. Lactate has shown prognostic value and risk stratification in critical patients^26^. Previous studies showed that lactate is significantly correlated with the prognosis of various shock states, including septic shock^27^, cardiogenic shock^28^ and traumatic shock^29^. AMI is the most common cause of cardiogenic shock^30^. Previous studies have shown that increased serum lactate in acute coronary syndrome (ACS) could predict the survival outcome^12, 31^. This is consistent with our findings. In this study, our study population consisted of critically ill patients with acute myocardial infarction, we found that with the increase of L/A ratio, lactate level increased, and the mortality of patients increased significantly. However, lactate has complex metabolic and clearance mechanisms^32, 33^, it is not easy to accurately identify a disease by using this indicator alone.

As the most abundant protein in plasma, serum albumin is widely used in clinical disease monitoring^34^. Studies have shown that hypoalbuminemia is not only associated with short-term mortality and new heart failure during hospitalization in patients with acute myocardial infarction^14^, but also independently predicts long-term cardiovascular outcomes. Meanwhile, there has a dose-response relationship between hypoalbuminemia and increased long-term all-cause mortality^35, 36^. This is also consistent with our findings that the L/A group with higher mortality rates had lower serum albumin levels. However, serum albumin levels can also be affected by a patient's blood glucose, nutritional status, and liver function^13^, so it also has certain limitations.

Although both plasma lactate and albumin have a connection to cardiovascular disease, previous studies demonstrated limited evidence about those systemic inflammatory biomarkers alone examining this reciprocal relationship to prognosis of AMI. Thus, a combined indicator L/A ratio has been proposed by more studies, which could reflect the state of oxygen supply and inflammation levels, and the combination of these two indicators can reflect the current pathophysiological state of patients more comprehensively than a single indicator from different mechanisms. Shin, Jikyoung's study^15^ demonstrated that the L/A ratio had a higher predictive value than lactate for 28-day mortality in patients with critical sepsis and Esra Cakir^16^ further demonstrated that the L/A ratio had a higher predictive value than either lactate or albumin alone for mortality in patients with sepsis in the ICU. According to this study, we discovered that L/A ratio was connected to 14-day, 28-day and 90-day all-cause mortality of AMI patients. Higher L/A ratios reflected increased lactate levels and decreased albumin levels.In addition, we used Cox proportional risk model and RCS model to analyze the connection between L/A ratio and the risk of AMI. The correlation between them is explained as follows.In the event of a severe AMI, the myocardium begins to work ineffectively due to insufficient oxygen uptake, leading to a decrease in myocardial extraction of lactic acid from the circulation^37^, as well as inducing the cell to preferentially undergo anaerobic glucose metabolism, which results in the production of lactic acid from pyruvate^38^. What’s more, the increase of lactate can participate in the process of inflammatory injury in the body, triggering the activation of a series of cell signaling pathways, so as to regulate the progression of inflammation^39^. Serum albumin has anti-inflammatory, antioxidant, anticoagulant and antithrombotic physiological properties, which is may be its potential role in cardiovascular disease^13^. It has been reported that inflammation has a negative effect on serum albumin levels^14^. Atherosclerosis is an inflammatory disease^40^, which can gradually progress to acute myocardial infarction^41^, which also lead to hypoalbuminemia. Our study demonstrates that L/A ratio is significantly associated with the prognosis in AMI patients, suggesting that this combined biomarker may capable to be better indicative of risk of AMI patients. Therefore, regarding the prognosis of individuals suffering from severe acute myocardial infarction, a significant emphasis on the L/A ratio has important clinical implications.

Our study also has some limitations. First of all, the MIMIC-III database is a large, single-center database with a large number of critically ill patients, which has the common limitations of a single-center database. Our results may not be fully applicable to intensive care units in other countries, but the patients included were from different ethnic groups and therefore are somewhat representative. Secondly, our study only included lactic acid and albumin data at the time of admission, without dynamic changes after admission. Meanwhile, due to the limitations of the database, it's possible that our model doesn't include more risk variables. Thirdly, the database lacks the position and number of stents in PCI, so we could not explore the link between the position as well as number of stents and prognosis of AMI. Fourthly, the database did not record the time of occurrence of AMI, so we didn’t analyzed the impact of time-laps on the L/A ratio in patients with AMI. Thus, greater evidence is needed to support our findings.

## Conclusions

The L/A ratio was substantially related to 14-day, 28-day and 90-day all-cause mortality in critical patients with AMI. Therefore, L/A ratio was capable of being used as a reliable predictor of prognosis in patients with AMI.

## Methods

### Source of data

The data sources is a large database called Medical Information Mart for Intensive Care III (MIMIC III) database. This free database contains information on various hospitalization categories from more than 40,000 patients who were older than 16 and admitted to Beth Israel Deaconess Medical Center's intensive care units (ICU) between 2001 and 2012^21^. To obtain data access, we successfully completed the Cooperative Institution Training Initiative exam and Cyber Training Program of the National Institutes of Health (NIH) which was to protect participants of human research. Due to patient's anonymity and the absence of protected information in the database, the ethics committee decided to waive informed consent.

### Inclusion and exclusion criteria

In this study, we comprised a total of 3,177 first time ICU admissions for AMI patients [using the International Classification of Diseases Version 9 (ICD-9) code]. There excluded 1,984 patients who were under 18 years of age, over 80 years of age or with an inexact age, 59 patients who didn't have lactate or albumin at admission.

### Data extraction

Data were collected from the MIMIC III database using Structured Query Language (SQL) with PostgreSQL (version 9.6). Demographic information (age, sex, ethnicity), Vital Signs (systolic blood pressure, diastolic blood pressure, mean blood pressure, heart rate, respiratory rate, body temperature), type of hospital admission (Elective, Emergency, Urgent), past medical history [AF, hypertension, diabetes, hyperlipidemia, COPD, AKI, ARDS, sepsis, CHF], laboratory indicators were measured multiple times within 24 h of admission, the first measurement was used [pH, SpO_2_, BE, AG, lactate, hemoglobin, platelet (PLT), WBC, albumin, BUN, Scr, glucose, sodium, potassium, PT, prothrombin time activity (PTA), oral medication on admission (aspirin, clopidogrel, beta blockers, diuretics, digitalis, statin), anti-diabetic therapy (the use of insulin and oral hypoglycaemic agents), cronary artery revascularization [(PCI, percutaneous transluminal coronary angioplasty (PTCA), CABG], SAPS II and SOFA were recorded. To minimize bias due to missing data, variables with more than 10% missing values were excluded from the final cohort. In fact, the missing values of indicators included in our study were less than 10%, such as body temperature, BE. We predicted the missing data using the multiple imputation method.

### Clinical outcomes

We obtained patient's clinical outcome and the specific time of death through the records of the Social Security Death Index, and the endpoints were 14-day, 28-day and 90-day all-cause mortality after ICU admission.

### Data analysis

Based on the tertiles of L/A ratio, the patients were divided into three groups : T1 group (L/A ratio<0.4063, *n*=379), T2 group (0.4063≤L/A ratio≤0.6667, *n* =379), and T3 group (L/A ratio>0.6667, *n* =376). Continuous variables that conform to the normal distribution are expressed as mean ± standard deviation. Continuous variables that are not normally distributed are expressed as median (interquartile range). Categorical variables are expressed as numbers (percentages). Independent sample T test or Mann-Whitney U test were used for continuous variables, while categorical variables were subjected to the Chi-square test. Then we established univariate and multivariate Cox proportional hazard models to test the correlation between the tertiles of L/A ratio and clinical outcomes ( the reference group was the first tertile). A variable of 0.05 was included in separate multivariate models of all-cause mortality at 14-day, 28-day and 90-day: Model 1, unadjusted; Model 2, involved age, sex, SBP and DBP; Model 3, involved variables in model 2 and hypertension, diabetes, hyperlipidemia, AF, COPD, CHF; Model 4 involved variables in model 3 and aspirin, clopidogrel, beta blockers, diuretics, digitalis and statin, insulin, oral hypoglycemic agents; Model 5 involved variables in model 4 and BUN, Scr, glucose, WBC, Hb, BE, and SpO_2_. At the same time, when L/A ratio was considered as a continuous variable, for more flexible modeling and visualizing the association between L/A ratio on admission and 14-day, 28-day and 90-day risk of mortality, we used the restricted cubic spline (RCS) curve. The cumulative incidence of 14-day, 28-day and 90-day all-cause mortality was calculated through performing Kaplan-Meier survival curves. Receiver operating characteristic curve (ROC) was used to compute the area under the curve (AUC) of the L/A ratio for predicting all-cause mortality. Meanwhile, we conducted subgroup analysis and presented it in the form of forest plot. A two-sided P <0.05 was considered statistically significant. All analyses were performed using R (R Foundation for Statistical Computing, Vienna, Austria).

### Ethical approval and consent to participate

The data we used was extracted from a publicly available critical care database-Medical Information Mart for Intensive Care III (MIMIC III, Version 1.4). The privacy of patients in MIMIC III were protected by using anonymized personal identifier. To protect the privacy of the participants, their identification information was concealed. Therefore, we did not need the specific consent procedures from our institutional ethics committee.

### Supplementary Information

Supplementary Information 1.

Supplementary Information 2.

Supplementary Information 3.

Supplementary Information 4.

Supplementary Information 5.

Supplementary Information 6.

Supplementary Information 7.

Supplementary Information 8.

Supplementary Information 9.

## Data Availability

This study analyzed publicly accessible datasets. This data can be found here: https://mimic.mit.edu/docs/.
