# Supplementary figures and images for: Association between lactate/albumin ratio and all-cause mortality in critical patients with acute myocardial infarction
Source: Sci Rep. 2023 Sep 20;13:15561. doi: 10.1038/s41598-023-42330-8 (PMC10511737; doi:10.1038/s41598-023-42330-8)

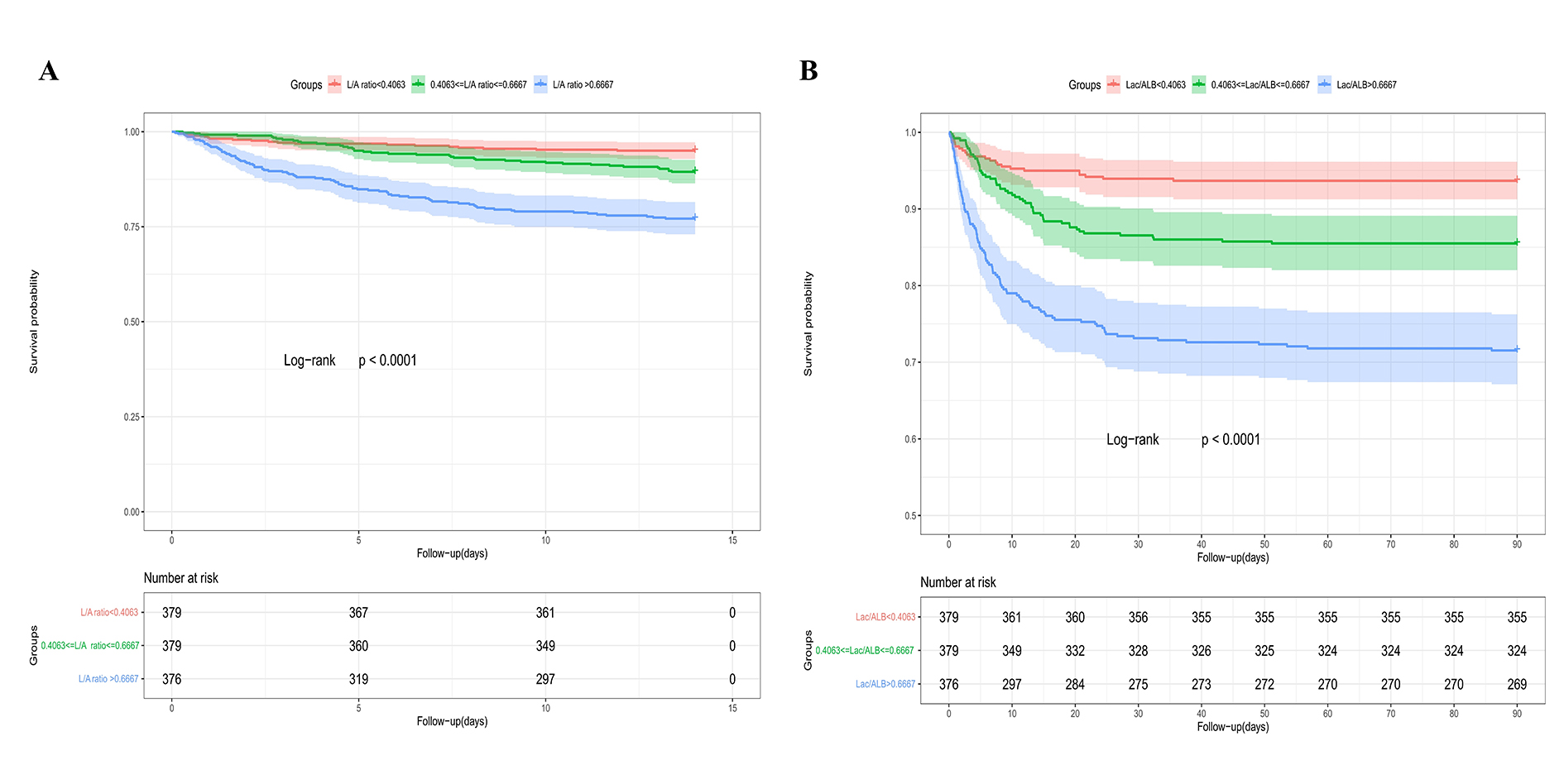

Supplement: Supplementary file 1 — Supplementary Information 1. [file 41598_2023_42330_MOESM1_ESM.jpg]

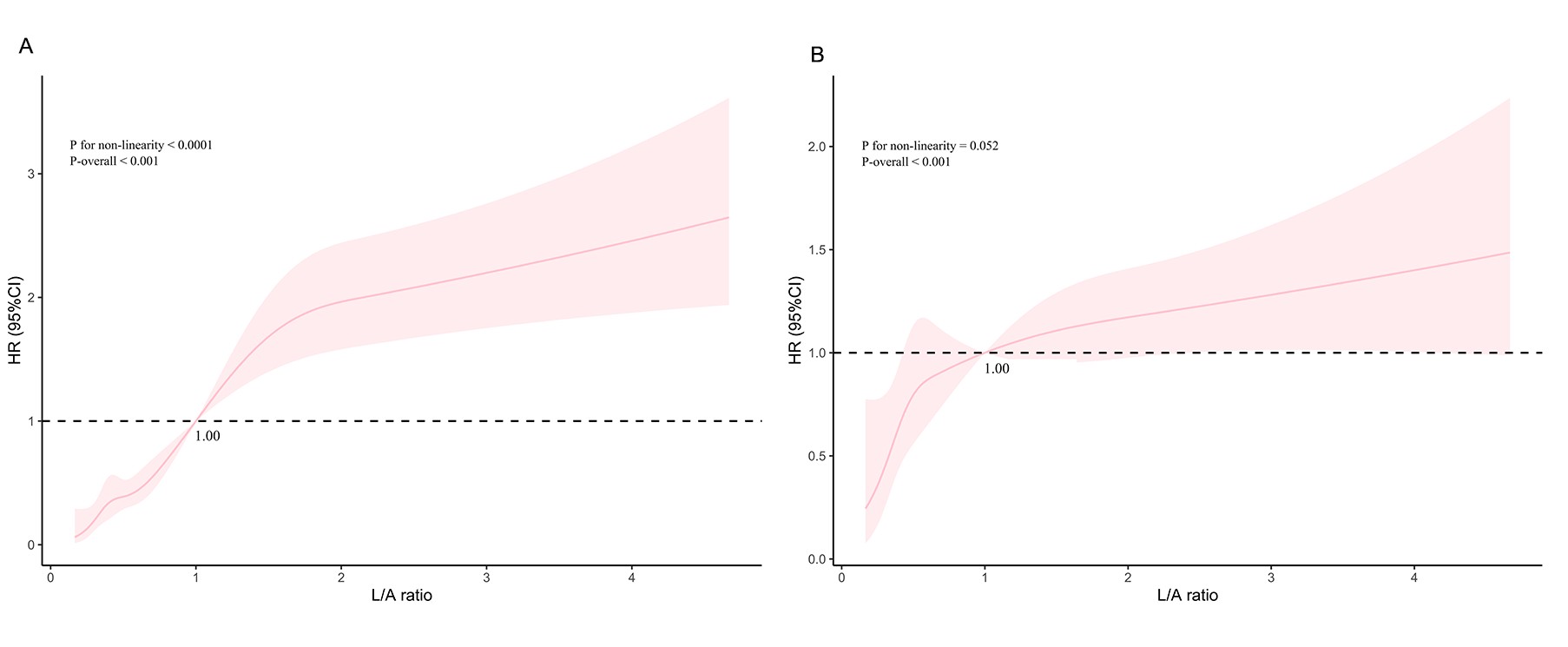

Supplement: Supplementary file 2 — Supplementary Information 2. [file 41598_2023_42330_MOESM2_ESM.jpg]

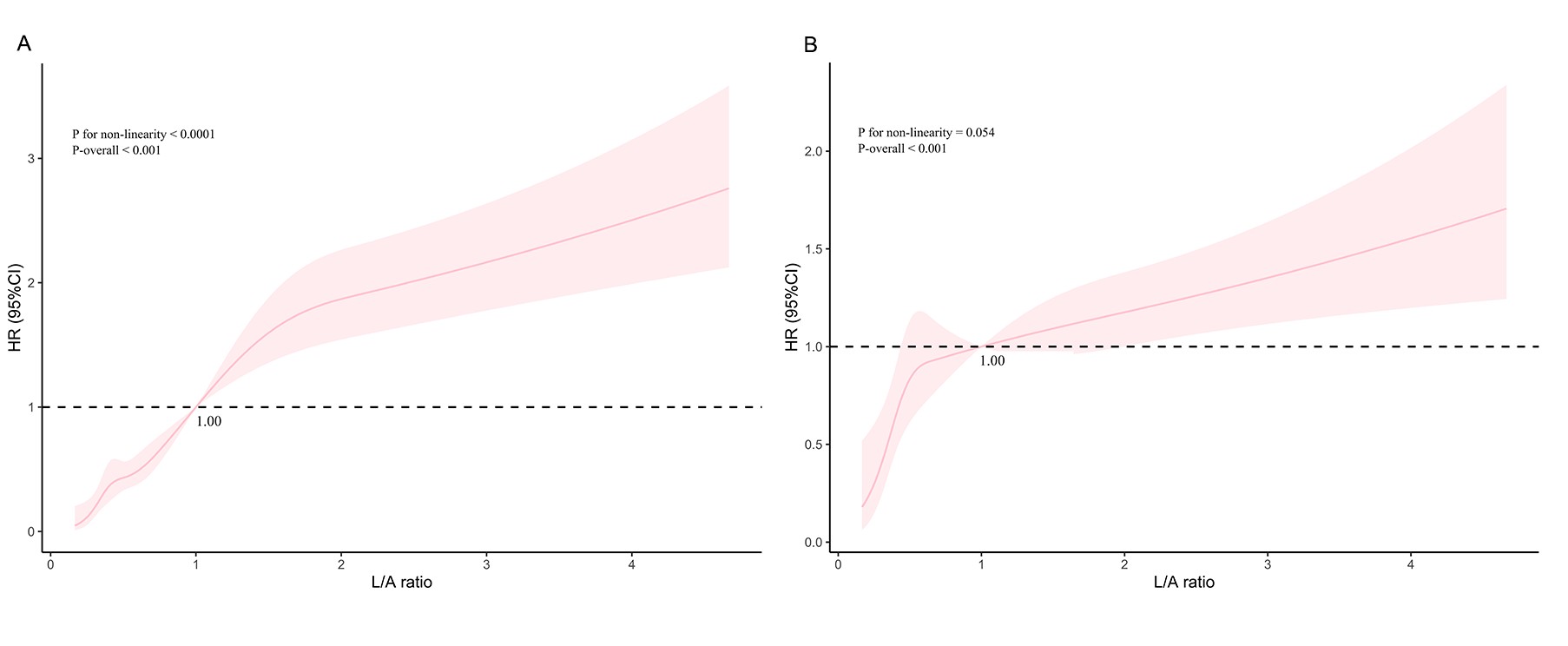

Supplement: Supplementary file 3 — Supplementary Information 3. [file 41598_2023_42330_MOESM3_ESM.jpg]

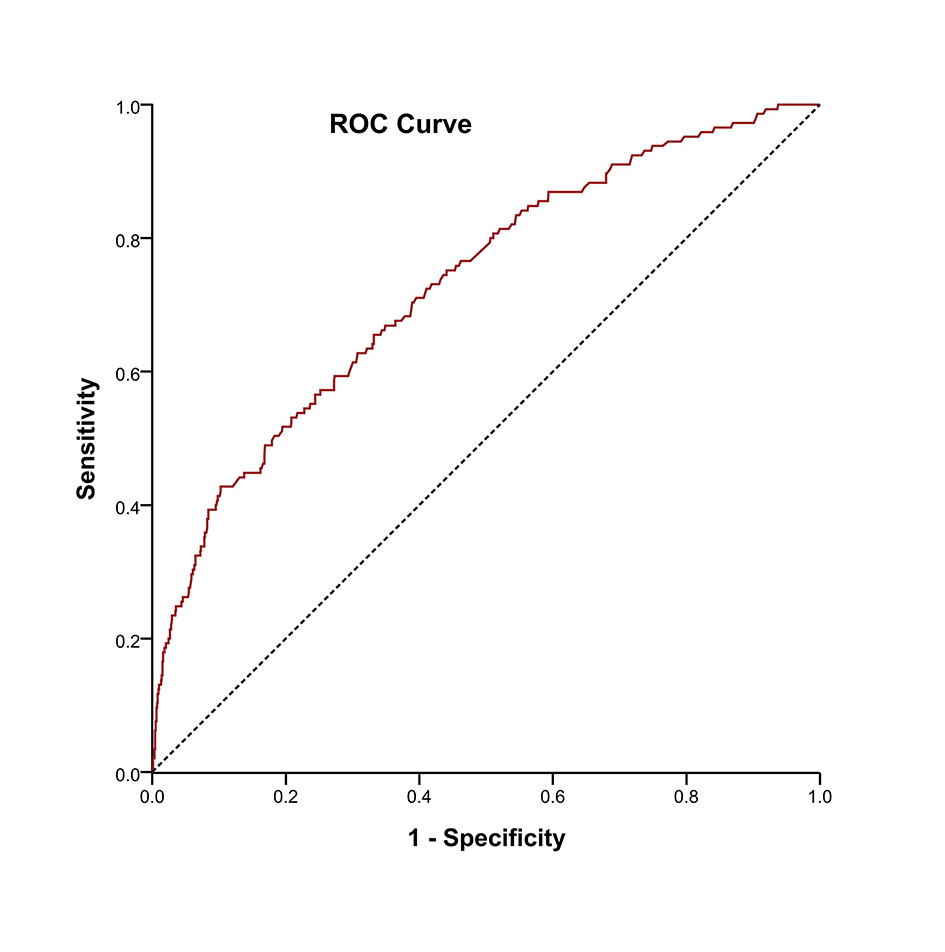

Supplement: Supplementary file 4 — Supplementary Information 4. [file 41598_2023_42330_MOESM4_ESM.jpg]

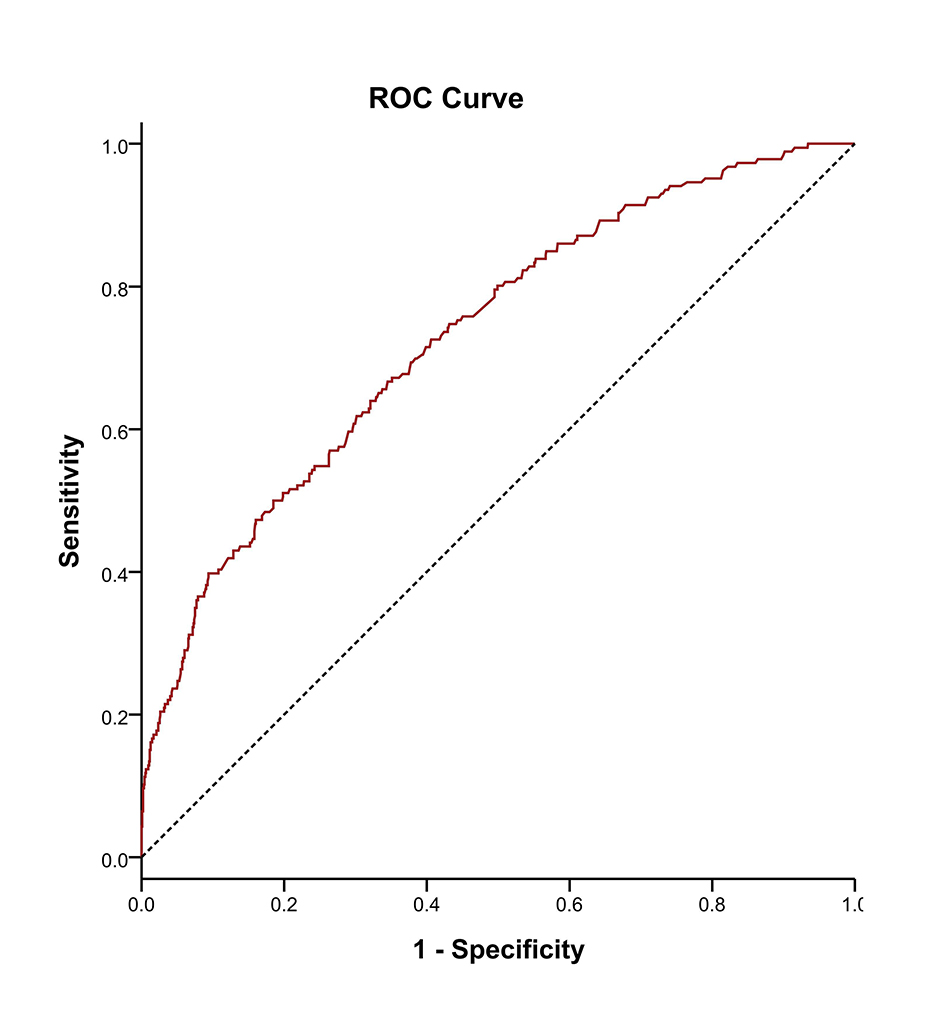

Supplement: Supplementary file 5 — Supplementary Information 5. [file 41598_2023_42330_MOESM5_ESM.jpg]
