# Supplementary material for: Association between lactate/albumin ratio and all-cause mortality in critical patients with acute myocardial infarction
Source: Sci Rep. 2023 Sep 20;13:15561. doi: 10.1038/s41598-023-42330-8 (PMC10511737; doi:10.1038/s41598-023-42330-8)
Supplement: Supplementary file 6 — Supplementary Information 6. [file 41598_2023_42330_MOESM6_ESM.docx]

**Supplementary Figure legends**

**Supplementary Figure 1.** Kaplan-Meier survival curve of 14-day all-cause mortality (A) and 90-day all-cause mortality (B) stratified by L/A ratio.

**Supplementary Figure 2.** The adjusted cubic spline model on the association between L/A ratio on a continuous scale and adjusted risk of 14-day all-cause mortality in patients with AMI. Crude hazard ratio (HR) and 95% CI for L/A ratio in 14-day mortality(A). Adjusted HR and 95% CI for L/A ratio in 14-day mortality (B). Adjusted variables included age, gender, SBP, DBP, hypertension, diabetes, hyperlipemia, AF, COPD, CHF, aspirin, clopidogrel, beta blockers, diuretics, digitalis, statin, insulin,

oral hypoglycemic agents, BUN, Scr, glucose, WBC, Hb, BE, SpO_2_.

**Supplementary Figure 3.** The adjusted cubic spline model on the association between L/A ratio on a continuous scale and adjusted risk of 90-day all-cause mortality in patients with AMI. Crude hazard ratio (HR) and 95% CI for L/A ratio in 90-day mortality (A). Adjusted HR and 95% CI for L/A ratio in 90-day mortality (B). Adjusted variables included age, gender, SBP, DBP, hypertension, diabetes, hyperlipemia, AF, COPD, CHF, aspirin, clopidogrel, beta blockers, diuretics, digitalis, statin, insulin, oral hypoglycemic agents, BUN, Scr, glucose, WBC, Hb, BE, SpO_2_.

**Supplementary Figure 4.** Receiver operating characteristic (ROC) curve for 14-day all-cause mortality.

**Supplementary Figure 5.** Receiver operating characteristic (ROC) curve for 90-day all-cause mortality.
