# Supplementary material for: Association between lactate/albumin ratio and all-cause mortality in critical patients with acute myocardial infarction
Source: Sci Rep. 2023 Sep 20;13:15561. doi: 10.1038/s41598-023-42330-8 (PMC10511737; doi:10.1038/s41598-023-42330-8)
Supplement: Supplementary file 7 — Supplementary Information 7. [file 41598_2023_42330_MOESM7_ESM.docx]

**Supplementary Table 1** Cox proportional hazard models for 14-day all-cause death.

| Variables | L/A ratio < 0.4063 | 0.4063≤L/A ratio≤ 0.6667 | L/A ratio > 0.6667 |
| --- | --- | --- | --- |
| Model 1^a^ | 1.000 (Ref.) | 2.127 (1.232-3.672) | 5.069 (3.084-8.331) |
| P value | - | 0.007 | ＜0.001 |
| Model 2^b^ | 1.000 (Ref.) | 2.116 (1.209-3.702) | 4.179 (2.497-6.992) |
| P value | - | 0.009 | ＜0.001 |
| Model 3^c^ | 1.000 (Ref.) | 2.144 (1.225-3.753) | 4.314 (2.576-7.223) |
| P value | - | 0.008 | ＜0.001 |
| Model 4^d^ | 1.000 (Ref.) | 1.662 (0.947-2.917) | 2.611 (1.544-4.413) |
| P value | - | 0.077 | ＜0.001 |
| Model 5^e^ | 1.000 (Ref.) | 1.636 (0.923-2.900) | 1.813 (1.041-3.156) |
| P value | - | 0.092 | 0.036 |

^a^ Model 1 Univariate model;

^b^Model 2 adjusted for age, gender, SBP, DBP;

^c^Model 3 adjusted for model 2 plus hypertension, diabetes, hyperlipemia, AF, COPD, CHF;

^d^Model 4 adjusted for model 3 plus aspirin, clopidogrel, beta blockers, diuretics, digitalis, statin, insulin, oral hypoglycemic agents;

^e^Model 5 adjusted for model 4 plus BUN, Scr, glucose, WBC, Hb, BE, SpO_2_.
