# Supplementary material for: Association between lactate/albumin ratio and all-cause mortality in critical patients with acute myocardial infarction
Source: Sci Rep. 2023 Sep 20;13:15561. doi: 10.1038/s41598-023-42330-8 (PMC10511737; doi:10.1038/s41598-023-42330-8)
Supplement: Supplementary file 8 — Supplementary Information 8. [file 41598_2023_42330_MOESM8_ESM.docx]

**Supplementary Table 2** Cox proportional hazard models for 90-day all-cause death.

| Variables | L/A ratio < 0.4063 | 0.4063≤L/A ratio≤ 0.6667 | L/A ratio > 0.6667 |
| --- | --- | --- | --- |
| Model 1^a^ | 1.000 (Ref.) | 2.352 (1.456-3.800) | 5.140 (3.301-8.004) |
| P value | - | <0.001 | <0.001 |
| Model 2^b^ | 1.000 (Ref.) | 2.313 (1.418-3.771) | 4.424 (2.804-6.980) |
| P value | - | 0.001 | <0.001 |
| Model 3^c^ | 1.000 (Ref.) | 2.279 (1.397-3.718) | 4.260 (2.697-6.627) |
| P value | - | 0.001 | <0.001 |
| Model 4^d^ | 1.000 (Ref.) | 1.876 (1.147-3.069) | 2.924 (1.835-4.659) |
| P value | - | 0.012 | <0.001 |
| Model 5^e^ | 1.000 (Ref.) | 1.934 (1.176-3.183) | 2.307 (1.426-3.733) |
| P value | - | 0.009 | 0.001 |

^a^ Model 1 Univariate model;

^b^Model 2 adjusted for age, gender, SBP, DBP;

^c^Model 3 adjusted for model 2 plus hypertension, diabetes, hyperlipemia, AF, COPD, CHF;

^d^Model 4 adjusted for model 3 plus aspirin, clopidogrel, beta blockers, diuretics, digitalis, statin, insulin, oral hypoglycemic agents;

^e^Model 5 adjusted for model 4 plus BUN, Scr, glucose, WBC, Hb, BE, SpO_2_.
